# Characterization of larval habitats of *Anopheles (Nyssorhynchus) darlingi* and associated species in malaria areas in western Brazilian Amazon

**DOI:** 10.1590/0074-02760240116

**Published:** 2024-10-07

**Authors:** Fátima dos Santos, Mingrui Xu, Lelys Bravo de Guenni, Ricardo Lourenço-de-Oliveira, Yasmin Rubio-Palis

**Affiliations:** 1 Fundação Nacional de Saúde-Funasa, Porto Velho, RO, Brasil; 2 University of Illinois, Champaign, USA; 3 Fundação Oswaldo Cruz-Fiocruz, Instituto Oswaldo Cruz, Rio de Janeiro, RJ, Brasil; 4 Universidad de Carabobo, Maracay, Venezuela

**Keywords:** *Anopheles* spp., climate, principal components, Poisson log-linear model, Negative Binomial log-linear model, malaria

## Abstract

**BACKGROUND:**

*Anopheles darlingi* is the most efficient vector of malaria parasites in the Neotropics. Nevertheless, the specificities of its larval habitats are still poorly known.

**OBJECTIVES:**

Characterize permanent larval habitats, and population dynamics of *An. darlingi* and other potential vectors in relation to climate, physicochemical variables, insect fauna and malaria cases.

**METHODS:**

A 14-month longitudinal study was conducted in Porto Velho, Rondônia, western Brazilian Amazon. Monthly, 21 permanent water bodies were sampled. Immature anophelines and associated fauna were collected, physicochemical characteristics, and climate variables were recorded and analyzed.

**FINDINGS:**

Five types of habitats were identified: lagoon, stream, stream combined with lagoon, stream combined with dam, and fishpond. A total of 60,927 anophelines were collected. The most abundant species in all habitats were *Anopheles braziliensis* and *An. darlingi*. The highest density was found in the lagoon, while streams had the highest species richness. Abundance was higher during the transition period wet-dry season. There was a lag of respectively four and five months between the peak of rainfall and the Madeira River level and the highest abundance of *An. darlingi* larvae, which were positively correlated with habitats partially shaded, pH close to neutrality, increase dissolved oxygen and sulphates.

**MAIN CONCLUSIONS:**

The present study provides data on key factors defining permanent larval habitats for the surveillance of *An. darlingi* and other potential vectors as well as a log-linear Negative Binomial model based on immature mosquito abundance and climate variables to predict the increase in the number of malaria cases.

*Anopheles* (*Nyssorhynchus*) *darlingi* Root is considered the most efficient vector of malaria parasites in the Neotropics,^1^ ^,^ ^2^ especially in the Amazon basin^3^ ^,^ ^4^ where over 90% of malaria cases in the Americas occur.^5^ The State of Rondônia (RO), in the southwestern Brazilian Amazon, has been historically a hot spot for malaria transmission, particularly in the municipality of Porto Velho (PVH).^6^ ^,^ ^7^ Between 2000 and 2021, 182,117 cases were reported in RO, 52% of which were reported for PVH.^8^ As in the rest of the Amazon, the causes for high malaria prevalence are diverse and complex, but notably deforestation for human settlements, agriculture, mining, and timbering together with human migration including cross-border malaria, parasite resistance to drugs, relapses of *Plasmodium vivax*, insecticide resistance and vector behavioral resistance are among those causes.^9^ ^,^ ^10^ ^,^ ^11^ ^,^ ^12^

Although *An. darlingi* has been associated with riverine forested areas,^13^ changes in land use have created appropriate conditions for the proliferation of this species throughout a vast area east of the Andes between 10º N and 20º S.^14^ ^-^ ^22^

Larval habitats of *An. darlingi* are diverse, and include natural water bodies such as streams, lagoons and lakes, and man created larval habitats such as fishponds, dams, abandoned gold mining dugouts, and brick pits.^23^ ^,^ ^24^ Despite the importance of identification and characterization of anopheline larval habitats for the design and implementation of control interventions, few studies have been conducted in the Amazon basin, particularly in Brazil.^25^ ^-^ ^31^ Moreover, longitudinal studies characterizing larval habitats and describing population dynamics of immature stages of *An. darlingi*, and other potential or secondary malaria vectors have not been conducted in the Brazilian Amazon.

The present study was conducted for 14 months with the goal of characterizing anopheline larval habitats and focusing on the population dynamics of *An*. *darlingi* in relation to climate, physicochemical variables, associated fauna and malaria cases in PVH.

## MATERIALS AND METHODS

*Study area* - The municipality of PVH, RO, (08º 07’S, 63º 39’W), Brazil, with an area of 34,091 km^2^. At the time of the study, the municipality accounted for 65 neighborhoods in the urban area and 446 localities in peri-urban and rural areas, with a population of 314,525 people.^32^ This area is located over the following geological formations: Jamari Complex, Serra de la Providencia Granites, Jací-Paraná Formation^33^ and the Rio Madeira Formation, composed of fluvial sediments beginning with a basal conglomerate with sandy and calcareous matrix, frequent manganese, and carbonaceous material.^34^ The physical relief has been classified as flat and gently undulating.^35^ The study area is in the Madeira River basin. The water level of the Madeira River may vary up to 21 m between February and April, down to 9 m between September and November.^36^ The Madeira River runs across PVH and influences the formation of natural and permanent water collections that are suitable larval habitats for anophelines all year.

The climate in PVH is characterized by high temperature and humidity, with a mean annual temperature of 26ºC, average relative humidity of 86%, and annual rainfall of 2,300 mm,^37^ ^,^ ^38^ and two well defined seasons: the rainy season (October to April), and a dry season (May to September) [Supplementary data (Table I)].

Although rate of deforestation has increased, the study area is mostly covered by Amazonian Forest, with trees of 30 to 50 m high represented by genera such as *Hevea*, *Bertholetia* and *Diniza*.^39^

For decades, PVH has been classified as an area of high malaria risk.^40^ Of the total number of malaria cases reported in the municipality during the study period (17,856), 74% were reported from rural areas and the remaining 26% from urban/peri-urban areas.^32^ The most prevalent malaria parasite species is *P. vivax* (88.7%), followed by *Plasmodium falciparum* (10.4%) and mixed infections (0.9%).^32^ Although the situation has improved in recent years, PVH was still among the six municipalities with the highest number of malaria cases in the country in 2020 (~ 6,000 cases); although, a lower risk (Annual Parasite Index < 10) was reported for 2023.^41^

*Climatological data* - Monthly air temperature, relative humidity and cumulative rainfall were obtained from the government climatological station located in the city of PVH.^37^

*Identification and selection of larval habitats* - It has long been known that the larval habitats of *An*. *darlingi* in the Brazilian Amazon are essentially large, deep and permanent water bodies.^3^ ^,^ ^23^ ^,^ ^42^ Thus, the urban and peri-urban areas of PVH were surveyed for large, deep and permanent water bodies. All potential habitats were then identified, inventoried, and evaluated against the following inclusion criteria for use in a longitudinal study: 1) located in an area with recently reported malaria cases, 2) without vector control interventions during the last 12 months, and 3) free of evident domestic or industrial pollution.

*Characterization of the aquatic environment* - The following characteristics were recorded once a month, between 7:30 and 9:30 am, at each larval habitat for 14 months (April 1998 - May 1999): water turbidity, presence of floating detritus (twigs, leaves, flowers, and fruits), water movement (subjectively accessed as stagnant, moderate and strong), and associated vegetation as emergent, floating or submersed according to the classification of Hess and Hall.^43^ The physical characteristics included water surface area (km^2^), depth (m), temperature (ºC) and sun exposure (%); the later subjectively estimated as shaded, partial, or totally exposed to sunlight. The LaMotte Company kit (Chestertown, MD, USA) was used for determining water chemistry: dissolved oxygen (DO), dissolved carbon dioxide (DCO_2_), silica (SiO_2_), calcium carbonate (CaCO_3_), ammonia (NH_4_), sulphates (SO_4_^-2^), nitrate (NO_3_^-^), phosphorus (P), and chlorides (Cl^-^) expressed in mg/L; the pH was measured using a Lutron pH-meter (model pH 206). The chemical variables were estimated as the mean number of the results for three samples of water from the same larval habitat at each sampling event.

*Larval sampling technique* - The standard form for characterization of larval habitats of the Brazilian National Malaria Control Program was used. Briefly, immature mosquitoes and associated fauna were sampled with a 350 mL dipper, and at least 85 scoops taken at each sampled site per month. All collected *Anopheles* larvae were counted according to the instars (L_1_, L_2_, L_3_ and L_4_) and pupae; the L_4_ stage were fixed in 80% ethanol for species identification and the pupae were placed in plastic containers and transported to the laboratory and reared to adults for species identification. Larvae were collected from the margins of the habitats as well as among floating vegetation. An inflatable boat was used to collect samples in the large and deep habitats.

*Species identification* - Collected larvae were treated with a 10% potassium hydroxide for cleaning the specimens, then transferred to a microscope slide and examined under an Olympus CX4 microscope at 400X (eye piece 10X and objective 40X). The keys by Consoli & Lourenço-de-Oliveira^42^ and Faran^44^ and Linthicum^45^ were used for the identification of fourth stage larvae and adults that emerged from collected pupae. The other aquatic insects collected were preserved in 80% ethanol and identified at the family level using the keys of Stehr^46^ and Chu & Cutkomp.^47^

*Data analysis* - Cross-correlations and time series analyses were conducted between the monthly anopheline species abundance and rainfall, the level of the Madeira River, relative humidity (% RH), air temperature and the number of malaria cases. These analyses were also used to determine the time lag between each pair of environmental variables, mosquito species, and malaria cases using R Studio.^48^

A log-linear Poisson model was used to model the response variable (each anopheline species and total mosquitoes) and the explanatory (environmental) variables, with selection based on the highest correlation value obtained.

To test the interaction between multiple factors such as time (months) and physicochemical water characteristics of the most common larval habitats (streams) on the response variables (taxa abundance data), the distance-based redundancy analysis (dbRDA) was used.^49^ The software CANOCO version 4.0 was used for all the analysis.^50^ The dbRDA is a non-parametric test used to determine the significance of individual factors in a multifactorial variance analysis for multiple variables using association measurements of the principal components analysis.^51^ The abundance and temporal distribution of aquatic insects (anopheline larvae and other insects) in relation to the larval habitat conditions (physicochemical variables) and the community structure were investigated by means of a partial ordination technique.^51^ The larval density of the most abundant anophelines species were related to environmental variables and natural predators (aquatics insects) using the dbRDA. The RDA is recommended for the analysis of sites representing short ecological gradients where a linear relationship between species and environmental factors is assumed.^50^ ^,^ ^52^ Additionally, the partial RDA was used to determine if variations in the response variables could be attributed to explicative variables. For performing the RDA analysis, three matrices were used: matrix Y (*Anopheles* species larvae density per month); matrix X (physicochemical water properties), and matrix W (families of aquatic insects collected). For each analysis, the statistical significance of the overall model (relationship between the set of variables and the taxa abundances) was tested using 999 Monte Carlo permutations tests and the value of the sum of all canonical eigenvalues was recorded.^50^ ^,^ ^51^

## RESULTS

**Fig. 1:**
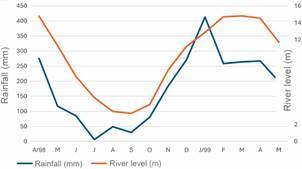
monthly rainfall (mm) and Madeira River level (m) between April 1998 and May 1999. Porto Velho, Rondônia, Brazil.

*Climatological data* - During the study period, the mean annual temperature was 26.4ºC, 68% mean annual relative humidity and 1,800 mm of rainfall.^37^ The level of the Madeira River varied from 3.32 m in September 1998 to 14.82 m in March 1999 [Fig. 1, Supplementary data (Table I)]. The time series data for the PVH climatological data for the period 1999-2021 reported a mean annual temperature of 26.1ºC, 84% relative humidity and annual rainfall of 2,216 mm,^53^ higher than that reported for the study period. This variation was mainly due to the strong El Niño Southern Oscillation event (ENSO) between June 1997 and June 1998.^54^

**Fig. 2:**
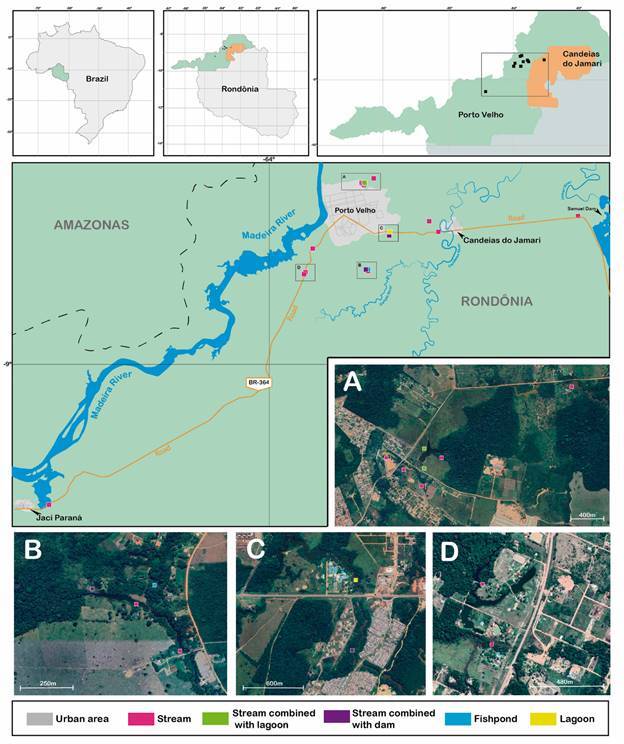
relative location of permanent larval habitats of anophelines in the Municipality of Porto Velho, Rondônia, Brazil.

*Characterization of the aquatic environment* - A total of 137 potential larval habitats corresponding to diverse hydrological types were identified in the study area, such as streams (*igarapés*), lagoons, dams, rain pools, ponds, swamps, excavations for the extraction of sand, and drainage channels. However, only 21 (16%) of them met the inclusion criteria and included: streams (N = 15), lagoon (1), fishpond (1), stream combined with lagoon (2) and stream combined with dam (2) (Fig. 2).

Streams: hydrological type most abundant in the study area; they are permanent with size varying from 3.45 ± 5.20 km^2^ and, 1.16 ± 0.84 m deep with moderate flow and shade produced by tall trees along the margins. The color of the water varies from dark to yellowish, while turbidity varies from slightly to completely turbid. Emergent vegetation such as *Montrichardia* spp. as well as floating plants like *Eichhornia* sp. and *Pistia* sp. were common in this type of larval habitat. The palm trees *Mauritia vinifera* (*buriti*) and *Orbignya oleifera* (*babaçu*) usually grew along the margins.

Lagoon: small (~ 800 m^2^) water body occurring in natural terrain depression originated from overflow of streams, 1.91 ± 0.61 m in depth; it was permanent with high diversity of emergent vegetation. The surrounding vegetation included tall trees and the banks were essentially covered by grass.

Fishpond: stagnant permanent water body created by construction of a dam in a stream for rearing the fish *Colossoma macropomum*; water is moderately turbid with a surface area of 3.4 km^2^, and 0.79 ± 0.23 m in depth. The pond margins were covered by grass.

Stream combined with lagoon: permanent and partially shaded water bodies, with a surface area of 1.05 ± 0.46 km^2^ and 1.05± 0.46 m in depth; moderate flow and turbidity varied from slightly turbid to clear.

Stream combined with dam: this permanent water body was created by damming a stream for domestic use. Throughout the study the water was slightly turbid, and with organic detritus present. The estimated surface area was 1.58 ± 0.32 km^2^ and 1.46 ± 0.89 m in depth; there was moderate water flow, and it was partially shaded.

Table I shows the physicochemical characteristics of each type of the 21 selected larval habitats.

**TABLE I:** Physicochemical characteristics of larval habitats and total number of immatures (L_1_-L_4_ + pupae) collected in Porto Velho, Rondônia, Brazil. April 1998 - May 1999

Variable	Stream (N = 15)	Lagoon (N = 1)	Fishpond (N = 1)	Stream + lagoon (N = 2)	Stream + dam (N = 2)
Silica (mg/L)	2.92 ± 2.94	2.5 ± 1.03	2.6 ± 0.5	2.23 ± 1.05	1.66 ± 4.15
Chlorides (mg/L)	6.52 ± 12.69	5.97 ± 7.97	6.9 ± 9.5	3.03 ± 7.07	4.09 ± 8.6
Dissolved oxygen (mg/L)	7.5 ± 2.57	8.84 ± 4.2	6.6 ± 1.96	7.5 ± 1.8	7.93 ± 1.65
Dissolved carbon dioxide (mg/L)	37.6 ± 37.8	63.9 ± 51.5	54.0 ± 32.8	63.0 ± 52.98	35.5 ± 27.81
Calcium carbonate (mg/L)	0.18 ± 0.44	0.6 ± 0.51	0.02 ± 0.0	0.12 ± 0.24	0.2 ± 0.25
Ammonia (mg/L)	1.0 ± 0.31	0.86 ± 0.35	1.0 ± 0.0	1.0 ± 0.0	1.2 ± 0.75
Sulphates (mg/L)	0.23 ± 0.25	0.2 ± 0.0	0.19 ± 0.001	0.42 ± 0.35	0.18 ± 0.041
Nitrate (mg/L)	0.34 ± 0.74	0.32 ± 0.24	0.19 ± 0.001	0.37 ± 0.55	0.21 ± 0.21
Phosphorus (mg/L)	0.38 ± 0.27	0.30 ± 0.30	0.52 ± 0.017	0.35 ± 0.28	0.41 ± 0.32
pH	7.18 ± 0.11	7.16^*^	7.24^*^	7.1^*^	7.28^*^
Temperature (ºC)	26.9 ± 1.3	27.13 ± 0.83	26.73 ± 0.88	26.8 ± 1.4	26.43 ± 1.7
Shade (%)	56.63 ± 13.39	71.0 ± 10.38	53.12 ± 15.58	55.46 ± 19.2	49.18 ± 17.8
Surface (km^2^)	3.45 ± 5.20	0.80	3.44	1.05 ± 0.46	1.58 ± 0.32
Depth (m)	1.16 ± 0.84	1.91 ± 0.61	0.79 ± 0.23	1.65 ± 0.29	1.46 ± 0.89
Water movement	Moderate	Slow	Stagnant	Moderate	Slow
Number of larvae + pupae (%)	40,630 (66.7)	4,116 (6.75)	1,686 (2.76)	6,154 (10.10)	8,341 (13.69)
Mean number per scoop x 10	11.6	19.5	8.7	13.6	17.5

N: number of larval habitats for each type; ^*^ Variation was too small to be considered.

*Species and anopheline abundance* - Between April 1998 and May 1999, 308 collections were performed in 21 habitats (Fig. 2). A total of 60,927 anopheline immatures (L_1_ to L_4_ and pupae) were collected (Table I). Although 66.7% were found in the 15 sampled streams, the highest mean number of larvae per scoop, regardless of developmental stage, was detected in the lagoon (19.5). Also, the higher mean number of identified immature forms (L_3_, L_4_ and pupae) collected per type of site was recorded for the lagoon (2,096) (Table II), followed by streams combined with lagoon (1,196.5), streams combined with dam (1,055), the fishpond (897) and stream (816.2). The number of larvae per scoop was higher for the lagoon (19.4/10), a slightly smaller number of immatures were collected in the streams combined with dam (17.5/10), followed by streams combined with lagoon, streams and the fishpond had the smaller frequency (8.7/10) [Table II, Supplementary data (Tables II-III)].

**TABLE II:** Number of *Anopheles* larvae identified in permanent aquatic habitats of Porto Velho, Rondônia, Brazil. April 1998 - May 1999

Species	Stream (N = 15)			Lagoon (N = 1)		Fishpond (N = 1)		Stream + lagoon (N = 2)			Stream + dam (N = 2)			TOTAL
	Total	Mean number per site	Mean number per scoop x10	Total	Mean number per scoop x10	Total	Mean number per scoop x10	Total	Mean number per site	Mean number per scoop x10	Total	Mean number per site	Mean number per scoop x10	
*An. braziliensis*	6,008	400.5	1.7	877	4.1	372	1.9	1,601	800.5	3.5	1,158	579	2.4	10,016
*An. darlingi*	3,877	258.5	1.1	588	2.8	154	0.8	436	218	1	472	236	1	5,527
*An. peryassui*	576	38.4	0.2	68	0.3	302	1.6	167	83.5	0.4	288	144	0.6	1,401
*An. triannulatus*	716	47.7	0.2	212	1.0	30	0.2	44	22	0.1	41	20.5	0.1	1,043
*An. albitarsis s.l*	537	35.8	0.2	218	1.0	0	0	82	41	0.2	21	10.5	0.0	858
*An. evansae*	380	25.3	0.1	64	0.3	38	0.2	44	22	0.1	120	60	0.3	646
*An. nuneztovari s.l.*	102	6.8	0.0	55	0.3	0	0	16	8	0.0	7	3.5	0.0	180
*An. oswaldoi s.l.*	31	2.6	0.0	0	0	0	0	0	0	0	0	0	0	31
*An. mattogrosensis*	10	0.7	0.0	6	0.0	1	0.0	2	1	0.0	3	1.5	0.0	22
*An. argyritarsis*	11	0.7	0.0	8	0.0	0	0	0	0	0	0	0	0	19
Total	12,248	816.5	3.5	2,096	9.9	897	4.6	2,393	1,196.5	5.3	2,110	1,055	4.4	19,744

Due to the difficulties in identifying early stages, only L_4_ and some L_3_ could be identified to species; representing 32.4% (19,744) of all specimens collected and comprising 10 species, with eight belonging to the subgenus *Nyssorhynchus* and two to the subgenus *Anopheles* (*i.e.*, *An. mattogrosensis* and *An. peryassui*). The most abundant species were *An. braziliensis* (50.7%) and *An. darlingi* (28.0%) (Table II). The streams were the type of habitat with higher species richness (10 species collected). Regarding the spatial distribution of species, *An. braziliensis*, *An. darlingi*, *An. peryassui*, *An. triannulatus s.l.*, *An. evansae* and *An. mattogrosensis* are considered generalist since they were collected at least once in all types of larval habitats. In contrast, *An. oswaldoi s.l.* was only collected in streams, while *An. nuneztovari s.l.* and *An. albitarsis s.l.* were never collected from the fishpond (Table II).

*Anopheles darlingi* was considerably more common in the lagoon, where 2.8 larvae (L_3_ and L_4_ larvae) were collected per scoop. Frequency of *An. darlingi* larvae per scoop was similar in the streams and stream combined with lagoon or dam (1 - 1.1), and less frequent in the fishpond (0.8).

**Fig. 3:**
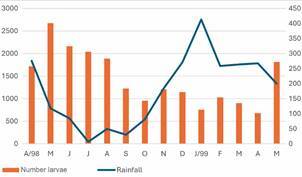
total number of *Anopheles* spp. immature stages identified collected monthly in all type of habitats in relation to rainfall (mm) in Porto Velho, Rondônia, Brazil. April 1998 - May 1999.

*Anopheline abundance in relation to environmental variables* - There was a major peak of the total identified immature stages of *Anopheles* spp. in May 1998, during the transition period from the rainy season to the dry season (Fig. 3).

**Fig. 4:**
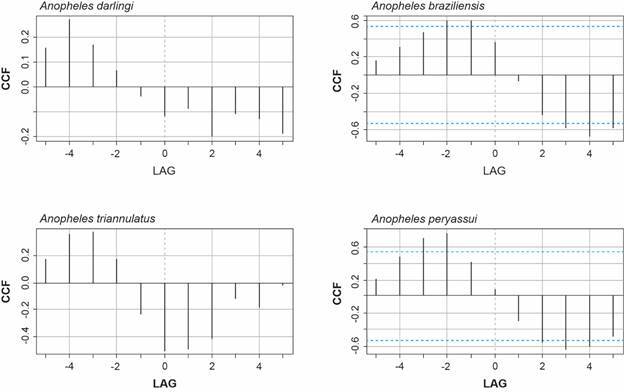
cross-correlation analysis between the variable rainfall and number of the four most abundant *Anopheles* species collected. Porto Velho, Rondônia, Brazil. April 1998 - May 1999.

Regarding the temporal distribution of the four most abundant species (*An. darlingi*, *An. braziliensis*, *An. peryassui* and *An. triannulatus s.l.*) in relation to rainfall, temperature, relative humidity and river level, a cross-correlation analysis was conducted to determine the time lag between each pair of environmental variables and mosquito species. Fig. 4 illustrates the cross-correlation relationship between the variable “Rainfall” and the population of the most abundant mosquito species. The plot clearly indicates that the cross-correlation function (CCF) line for rainfall, with lags of one and two months, surpasses the threshold for statistical significance, indicating a significant association between the two variables, particularly for *An. braziliensis* and *An. peryassui*.

Additionally, the ‘lag2.plot’ function from the Applied Statistical Time Series Analysis (astsa) R library^48^ was used to display the precise values of the correlations for the four most abundant species and the climate lagged variables.

To model the response of anopheline abundance to environmental variables, five Generalized Linear Models (GLM) function with a Poisson family were developed, each model focused on each of the four most abundant species (*An. braziliensis*, *An. darlingi*, *An. triannulatus s.l.* and *An. peryassui*) and the total number of mosquito immatures collected monthly and for the explanatory variables, three environmental variables were selected based on the highest correlation value obtained from the ‘lag2.plot’.

The log-linear Poisson model for the number of immature mosquitoes can be expressed as:

log(E(Y(t))) = β0 + β1 Rain(t-k1) + β2RH(t-k2) + β3RL(t-k3)

Where *E*(*Y*(*t*)) is the expected value of the response variable (number of mosquitoes for a given species) at time which is assumed to have a Poisson distribution; Rain, RH, and RL are respectively Rainfall (mm), Relative humidity (%) and River level (m) at lags *t*-*k*_*1*_ , *t*-*k*_*2*_ and *t*-*k*_*3*_ which are the lagged predictors in the model. Model coefficients *β*_*i*_ ,*i* = 0,…,3 are estimated by maximum likelihood (ML) using the R function *glm*. Lags *t*-*k*_*1*_ *, t*-*k*_*2*_ and *t*-*k*_*3*_ vary with mosquito species.

Table III shows the significant predictors (climate lagged variables) for each *Anopheles* species. Rainfall (mm) and River level (m) are significant (p < 0.05) in all models. However, relative humidity (% RH) was found to be significant in all models except for *An. darlingi* and *An. triannulatus s.l*.

**TABLE III:** P-values for the lagged environmental predictor variables of the monthly abundance of the four most abundant *Anopheles* species in Porto Velho, Rondônia, Brazil. April 1998 - May 1999

Variables	*An. darlingi*	*An. braziliensis*	*An. triannulatus*	*An. peryassui*	Total mosquitoes
Rainfall (mm)	0.0417 (lag = 4)	p < 0.01 (lag = 2)	p < 0.01 (lag = 3)	p < 0.01 (lag = 3)	p < 0.01 (lag = 2)
RH (%)	0.1108 (lag = 1)	p < 0.01 (lag = 1)	0.126 (lag = 3)	p < 0.01 (lag = 2)	p < 0.01 (lag = 2)
River level (m)	p < 0.01 (lag = 5)	p < 0.01 (lag = 1)	p < 0.01 (lag = 3)	p < 0.01 (lag = 2)	p < 0.01 (lag = 1)

*Relation of anophelines abundance, environmental variables, and malaria cases* - To model the malaria cases in response to anophelines abundance and rainfall, relative humidity and river level (explanatory variables), a log-linear Negative Binomial model was developed to address overdispersion.

The number of malaria cases can be modeled as:

log(E(Z(t)) = γ0 + γ1MN(t-k) + γ2Rain(t-4) + γ3RH(t-5) + γ4RL(t-4)

Where *E*(*Z*(*t*) is the expected number of malaria cases at time *t*; MN, Rain, RH, and RL are respectively Mosquito numbers, Rainfall (mm), Relative humidity (%) and River level (m) at lags at lag *t*-*k*, *t*-4, *t*-5 and *t*-4. In this case, a negative binomial distribution is assumed for the response variable *Z*(*t*). Model coefficients *γ*_*i*_ ,*i*=0,…,4 are estimated by ML using the R function *glm*. The value of lag t-k varies with mosquito species.

In all malaria models, the variables “Rainfall” and “River level” lagged by four months, while “relative humidity” lagged by five months. The selection of lagged values for different mosquito species was based on the outcomes obtained from the cross-correlation functions.

The p-values for the explanatory variables indicate that “relative humidity” is significant (p < 0.01) when the species “*darlingi*” is present in the model. In the model with “total mosquitoes”, “river level” becomes significant (p = 0.0237). However, in other models, no significant variables were found.

We also conducted diagnostic tests for the model using “*darlingi*” and “total mosquitoes,” employing deviance-based goodness-of-fit tests and residual plots. As the p-values exceeded the significance level of 0.05, we can confidently conclude that the models fit the data well. Furthermore, the residual plots exhibited a random pattern in the distribution of data points, further supporting the adequacy of the model fit.

*Associated fauna* - Together with immature stages of mosquitos of the genus *Anopheles*, 13,779 aquatic insects belonging to the orders Coleoptera, Diptera, Ephemeroptera, Heteroptera and Odonata were collected (Table IV). The most abundant were immature stages of Diptera belonging to the subfamily Culicinae (6,617). When considering the density of aquatics insects by habitat type in terms of mean number collected per scoop, Coleoptera and Heteroptera showed the higher density in the lagoon; Diptera and Ephemeroptera in the stream combined with lagoon, while Odonata density was higher in the fishpond. The lowest density of all orders was found in the streams. Characidae fishes were frequently observed in all types of habitats. Among the aquatic insects collected there were important predators of mosquito larvae such as members of the families Dytiscidae (Coleoptera),^55^ Belostomatidae, Corixidae, Nepidae and Notonectidae (Heteroptera),^56^ ^,^ ^57^ ^,^ ^58^ ^,^ ^59^ Libellulidae y Coenagrionidae (Odonata).^57^ ^,^ ^60^ ^,^ ^61^ ^,^ ^62^ ^,^ ^63^ The higher density of aquatic insects besides *Anopheles* was also recorded in May 1998 during the transition period between rainy and dry season showing a similar pattern as anopheline larval abundance.

**TABLE IV:** Number of aquatic insects (order and family) collected in association with anophelines in permanent larval habitats of Porto Velho, Rondônia, Brazil. April 1998 - May 1999. Mean numbers collected per site are shown between brackets

Insects	Stream	Lagoon	Fishpond	Stream + lagoon	Stream + dam	TOTAL
Order/Family	(N = 15)	(N = 1)	(N = 1)	(N = 2)	(N = 2)	
Coleoptera						
Dytiscidae^*^	220 (14.67)	15	14	43 (21.5)	56 (28)	348
Noteridae^*^	203 (13.53)	40	2	74 (37)	40 (20)	359
Scirtidae	44 (2.93)	7	12	48 (24)	7 (3.5)	118
Total	467 (31.13)	62	28	165 (82.5)	103 (51.5)	825
Mean number/scoop x100	0.089	2.93	1.45	1.82	1.08	
Diptera						
Ceratopogonidae	15 (1.0)	0	8	0	0	23
Chironomidae	18 (1.2)	0	0	0	0	18
Culicinae	6,517 (434.47)	63	142	910 (445)	126 (63)	7,758
Total	6,550 (436.67)	63	150	910 (445)	126 (63)	7,799
Mean number/scoop x100	1.25	2.98	7.76	9.81	1.32	
Ephemeroptera						
Baetidae	732 (48.8)	28	11	214 (107)	75 (37.5)	1,060
Caenidae	428 (28.53)	20	13	21 (10.5)	17 (8.5)	499
Tricorythidae	0	5	0	0	0	5
Total	1,160 (77.33)	53	24	235 (117.5)	92 (46)	1,564
Total number per scoop x100	0.22	2.51	1.24	2.59	0.97	
Heteroptera						
Belostomatidae^*^	227 (15.13)	17	0	99 (49.5)	53 (26.5)	396
Corixidae^*^	1,818 (121.2)	138	61	32 (16)	109 (54.5)	2,158
Gerridae^*^	1 (0.07)	0	0	0	1 (0.5)	2
Hydrometridae^*^	0	0	0	3 (1.5)	6 (3.0)	9
Naucoridae^*^	31 (2.07)	0	0	0	2 (1.0)	33
Nepidae^*^	3 (0.02)	0	0	0	0	3
Notonectidae^*^	539 (35.93)	33	20	43 (21.5)	45 (22.5)	680
Pleidae^*^	10 (0.67)	2	0	0	0	12
Veliidae^*^	38 (2.53)	4	2	7	2 (1.0)	53
Total	2,667 (177.8)	194	83	184 (92)	218 (109)	3,346
Mean number/scoop x100	0.51	9.18	4.3	2.03	2.29	
Odonata						
Coenagrionidae^*^	126 (8.4)	4	13	14 (7.0)	17 (8.5)	174
Libellulidae^*^	36 (2.4)	0	0	25 (12.5)	10 (5.0)	71
Total	162 (10.8)	4	13	39 (19.5)	27 (13.5)	245
Mean number/scoop x100	0.031	0.19	0.67	0.43	0.28	

^*^ Predators of mosquito larvae.

**Fig. 5:**
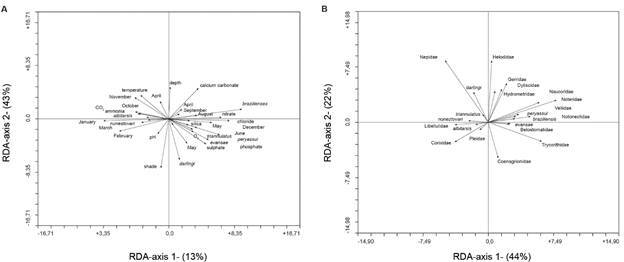
(A) Correlation biplots based on distance-based redundancy analysis (dbRDA) ordination of environmental variables, months, and *Anopheles* species in streams. Porto Velho, Rondônia, Brazil. April 1998 - May 1999. Vectors (arrows) point in the direction of increasing values for the respective variables; longer vectors indicate stronger correlations between variables scores and axes. (B) Correlation biplots based on dbRDA ordination of aquatic insects and *Anopheles* species in streams. Porto Velho, Rondônia, Brazil. April 1998 - May 1999. Vectors (arrows) point in the direction of increasing values for the respective variables; longer vectors indicate stronger correlations between variables scores and axes.

*Interaction between physicochemical water parameters, time (months), abundance of Anopheles species and aquatic insects* - Throughout the study, the water temperature within and between larval habitats did not show significant differences; thus, this variable was not considered in further analysis. The dbRDA of the *Anopheles* species in relation to environmental variables showed different temporal distributions and associations with the different physical and chemical variables in the streams, which were the most common larval habitats (Fig. 5A). The RDA ordination of variables in relation to *Anopheles* species abundance reflected the difference in species compositions among months (Fig. 5A). The first axis explains 13% of total variance in species distribution and abundance and the second axis explains 43%. Overall, *An. braziliensis*, *An. darlingi*, *An. peryassui*, *An. albitarsis s.l.*, *An. triannulatus s.l.*, *An. evansae* and *An. nuneztovari s.l.* were well-represented in the ordination diagram, with more than 12% of the variance accounted for the significance. Although this value might seem low, this type of analysis is highly informative.^50^ The diagram summarizes the relation of anopheline species with time (months) and indicates the estimated localization of maximum abundances and optimal physicochemical conditions for each species. The diagram shows two groups of species separated by the first axis: one group to the right comprising *An. darlingi*, *An. evansae*, *An. triannulatus s.l.*, *An. peryassui* and *An. braziliensis*, and the other group to the left comprised by *An. nuneztovari s.l.* and *An*. *albitarsis s.l*.

*Anopheles darlingi* is positively correlated with shaded larval habitats, pH close to neutrality, increased DO and sulphates during May (rainy-dry transition period); a similar pattern was shown by *An. evansae* and *An. triannulatus s.l.*, although in less shaded larval habitats. On the other hand, *An. darlingi* is negatively correlated with increased water temperature and depth, increased ammonia, and dissolved carbon dioxide.

*Anopheles peryassui* is correlated with the period of less rainfall in June and higher phosphates and chlorides. *An. albitarsis s.l.* and *An. nuneztovari s.l*. show contrasting behavior; *An. albitarsis s.l*. is correlated with October (dry-rainy transition period), less shaded habitats with higher dissolved carbon dioxide and ammonia, while *An. nuneztovari s.l*. is correlated with the months January, February, and March (rainy season), shallow and sun exposed larval habitats with lower dissolved carbon dioxide and ammonia, and pH closed to neutrality. *An. braziliensis*, the most abundant species, was positively correlated with the month of April (beginning of the rainy-dry transition period), deep larval habitats with increased concentration of nitrates and calcium carbonate, while this interaction decreased during August and September [Supplementary data (Table IV)]. Due to total loss of the primary data, it was not possible to conduct similar analyses among and between different types of larval habitats.

The ordination of *Anopheles* species in relation to aquatic insects is shown in Fig. 5B. The first axis explains 44% of the variance in species abundance and the second axis 22%. In the upper left-hand side of the figure, we found that *An. darlingi* is positively correlated with Nepidae (Heteroptera), and negatively correlated with Libellulidae (Odonata), Corixidae and Pleidae (Heteroptera); a similar association with these insect families was found for *An. triannulatus s.l*. and *An. nuneztovari s.l.* but significantly less strong than that observed for *An. darlingi*. Contrasting results were observed for *An. albitarsis s.l.*, which is positively correlated with these families. *An. braziliensis* and *An. peryassui* are correlated with Notonectidae, Veliidae, Hydrometidae, Naucoridae and Gerridae (Heteroptera), and Dytiscidae and Noteridae (Coleoptera); although negatively associated with Belostomatidae (Heteroptera), Trycorithidae (Ephemeroptera), and Coenagrionidae (Odonata). *An. evansae* is the only species correlated positively with members of these predatory families [Supplementary data (Table V)].

## DISCUSSION

*Anopheles darlingi* has a wide range of distribution in the Neotropics, from southern Mexico to northern Argentina^13^ and is the most efficient vector of malaria parasites in South America in terms of high anthropophilic habits and its capacity to transmit all three parasite species *P. falciparum*, *P. vivax* and *P. malariae*.^3^ ^,^ ^64^ ^,^ ^65^ ^,^ ^66^ The females of this species may oviposit in a wide variety of natural and man-made water bodies, permanent or temporal.^4^ ^,^ ^13^ However, its main larval sites are usually permanent, large and deep-water bodies like rivers, streams, lakes and lagoons. Human activities have also created water bodies that are colonized by *An. darlingi* and associated species, such as dams, fishponds, ditches, clay pits, ponds, abandoned gold mines and containers.^3^ ^,^ ^4^ ^,^ ^13^ ^-^ ^15^ ^,^ ^18^ ^-^ ^20^ ^,^ ^23^ ^,^ ^24^ ^,^ ^27^ ^,^ ^30^ ^,^ ^42^ ^,^ ^67^ ^-^ ^71^

Tremendous efforts have been made to reduce the incidence of malaria in the Brazilian Amazon, particularly in Rondônia.^7^ ^,^ ^64^ Nevertheless, the persistence of transmission might be due to resistance of the parasites to the available drugs,^72^ ^,^ ^73^ and the biting (partially exophagic) and resting behavior (exophilic) of populations of *An. darlingi* and other secondary vectors present in the area that escape interception either by long lasting insecticide treated nets (LLINs) and/or insecticide residual spraying (IRS).^22^ ^,^ ^23^ Studies conducted in urban and peri-urban areas of PVH have shown that *An. darlingi* females are the most abundant species with a biting peak outdoors between 1800 and 2000 hrs.^22^ ^,^ ^74^ ^,^ ^75^ These results suggest that traditional vector control measures are less effective, and alternative and/or combined interventions are necessary. Therefore, WHO^76^ has indicated that supplementary interventions based on immatures control are effective in reducing vector density and malaria transmission where larval habitats of the principal malaria vector are few, fixed and findable, and where larval control is both feasible and cost-effective. The design and implementation of cost-effective larval source management (LSM) demands a profound knowledge of the ecology and population dynamics of vector species in larval sites.

The present study provides base line data to implement a surveillance program, an essential activity for vector control interventions and evaluation of their impact on malaria transmission. Five types of permanent anophelines larval habitats were identified and characterized: lagoon, stream, stream combine with lagoon, stream combine with dam, and fishpond. The species richness was higher in streams (10 species), and fishponds the least. *An. darlingi*, *An. braziliensis*, *An. triannulatus s.l.*, *An. peryassui*, *An. evansae*, and *An. mattogrosensis* were collected, at least once, in all types of permanent water bodies; other species, although collected less frequently, were more specific. *An. nuneztovari s.l.* was not collected in the fishpond, while it was frequently collected (positivity index = 90.4%) in fishpond habitat in Amapá.^30^ These contrasting results might possibly be due to the fact that *An*. *nuneztovari s.l*. from Amapá may be a different species from the species present in PVH. As it has been shown, using morphological and molecular markers, *An. nuneztovari* is a complex of four species: *An. nuneztovari s.s*., *An. goeldii*, *An. dunhami* and *An. jamariensis.* ^77^ ^,^ ^78^ ^,^ ^79^ ^,^ ^80^ ^,^ ^81^ Probably the species collected in Amapá in fishponds by Barbosa & Scarpassa^30^ is *An. goeldii*, while the species collected in the present study is the recently described species *An. jamariensis* collected in the municipality of Monte Negro, Rondônia.^81^ Similarly, *An. albitarsis s.l*. was never collected in the fishpond in PVH, but it was in Amapá^30^ and Amazonas.^20^ *An. albitarsis* is a complex of at least 10 species of which only five have been formerly described: *An. albitarsis sensu stricto*, *An. deaneorum*, *An. marajoara*, *An. oryzalimnetes*, and *An. janconnae*.^82^ ^,^ ^83^ ^,^ ^84^ ^,^ ^85^ *An. deaneorum* and *An. oryzalimnetes* have been reported from Rondônia;^82^ ^,^ ^85^ ^,^ ^86^ based on morphological characters, Meireles et al.^22^ and Rodrigues et al.^87^ identified adults collected in PVH, as *An. deaneorum*. It is not possible to establish the species identity of the mosquitoes of the Albitarsis complex collected during the present study. *An. oswaldoi s.l*., probably *An. konderi* ^22^ ^,^ ^87^ was restricted to streams and with very low frequency. *An. albitarsis s.l*., *An. nuneztovari s.l.* and *An. oswaldoi s.l*. have been confirmed as vector of malaria parasites in different Amazonian states in Brazil [see Carlos et al.^64^ for a complete revision], as well as in Colombia^88^ ^,^ ^89^ ^,^ ^90^ and Venezuela,^91^ ^,^ ^92^ ^,^ ^93^ ^,^ ^94^ hence it is important that the surveillance program in PVH includes morphological and molecular identification of species belonging to these species complexes.

In general, the most productive larval habitat in terms of the mean number of larvae per scoop was the lagoon (Table I). The same was observed for *An. darlingi* which was more than twice as frequent there as in the other four habitat types (Table II). Similar results have been reported in Ameridian sites in Roraima and Amazonas,^24^ as well as in southern Venezuela.^14^ ^,^ ^18^ ^,^ ^19^ ^,^ ^69^ In terms of larval habitats, *An. darlingi* is a generalist species.^4^ ^,^ ^13^ The described physicochemical characteristics of water bodies within its range of distribution and types of most productive larval habitats vary. In the present study, the most distinctive characteristics of the lagoon in relation to the other types of water bodies is the higher percentage of shaded surface, depth, and DO. *An. darlingi* was frequently associated with the presence of *An. braziliensis*, *An. triannulatus s.l.* and *An. albitarsis s.l.* (Table II). *An. braziliensis* was the most abundant species in all larval habitats, particularly in the lagoon. These results highlight the heterogenous environmental conditions that determine the spatial distribution of anophelines species for the same type of larval habitat. In the Yanomami areas of Amazonas and Roraima, Sánchez-Ribas et al.^24^ reported that the most abundant species in lagoons was *An. oswaldoi s.l*. followed by *An. darlingi*; similar results were reported by Rubio-Palis et al.^14^ in a Yanomami area of southern Venezuela, while Moreno et al.^18^ ^,^ ^69^ ^,^ ^95^ reported that in lagoons in gold mining areas of southern Venezuela, *An. triannulatus* followed by *An. albitarsis s.l*. were the most abundant species.

Regardless of the type of water body, there are some physicochemical characteristics that might be important determinants for the development of *An. darlingi* such as partial shade, pH, depth, and DO. It has been reported that *An. darlingi* is not present in deep shaded forests where the most abundant mosquitoes belong to the genus *Culex*,^96^ ^,^ ^97^ ^,^ ^98^ nor in open savannahs in northern Roraima,^99^ Suriname^4^ and southern Venezuela.^18^ *An. darlingi* is associated with sites of human interventions such as deforestation for agriculture, cattle ranching, urban development, forestry and mining, where the remaining forested areas and the transition zone (secondary forest-shrub) provides optimal ecological conditions for the colonization of permanent or temporal water bodies with shaded areas above 45% in Brazil, ^27^ ^,^ ^70^ Colombia (68), Peru,^15^ Suriname,^100^ Venezuela,^14^ ^,^ ^18^ ^,^ ^19^ and Belize.^101^ Furthermore, Barros & Honório^70^ have shown that *An. darlingi* larvae are found in areas with decreased luminosity compared to sunlit areas within the same water body.

The pH of water is an important determinant for the presence and abundance of mosquitoes as it has been demonstrated that it is directly related to cell functioning and acts on the permeability of the cell membrane.^102^ In general, anophelines are found in water bodies with a pH around 7, as reported in the present study and inclusive for *An. darlingi*. Indeed, *An. darlingi* and associated species were not present in fishponds and streams in Amapá at pH between 5.0 and 5.91.^30^ Curiously, Rufalco-Moutinho et al.^27^ collected *An. darlingi* in natural larval habitats 100 times more acidic (pH = 5.28) than man-made habitats (pH = 7.12), while this species has been found in natural water bodies like lagoons, swamps, and streams of southern Venezuela, at pH between 10 to 1,000 times more acidic (pH = 4.9-6.8).^14^ ^,^ ^18^ ^,^ ^69^ The pH in water bodies is related to the characteristics of the bedrock, soil composition, vegetation, debris, and other elements present and often due to human activities such as agricultural practices (slash and burn, fertilizers), and cattle ranching.^103^ ^,^ ^104^ These findings emphasize a need to characterize the larval habitats in particular environments where malaria is endemic.

Although anophelines depend on atmospheric oxygen to breathe, the concentration of DO in larval habitats is an indication of water quality and its potential for the development of immature stages. The reported values of DO in the present study are considered high and adequate for the development of anophelines, providing conditions for the proliferation of algae and other microorganisms that support the diet of mosquito larvae.^105^ *An. darlingi*, *An. triannulatus s.l.*, *An. evansae*, and *An. peryassui* are positively associated with high concentrations of DO in streams (Fig. 5A) and negatively associated with *An. albitarsis s.l*. and *An. nuneztovari s.l*. The values of DO have been considered a determinant factor for the presence of anopheline species in larval habitats showing either positive or negative relationship. Arcos et al.^20^ found a negative correlation between DO and *An. darlingi* in fishponds in Manaus, while in Amapá *An. darlingi* was not associated with DO but *An. nuneztovari s.l.* and *An. triannulatus* were most affected by it.^30^ In Ecuador, *An. punctimacula* was positively associated with DO, and no association was found for *An. albimanus*, *An. pseudopunctipennis* and *An. oswaldoi s.l*.,^106^ whereas *An. albimanus* is associated to low levels of DO in Mexico and Belize.^107^ ^,^ ^108^ ^,^ ^109^

*Anopheles darlingi*, as well as associated species, was less frequent in the fishpond, contrasting these results with more recent studies in Amazonas,^20^ Acre,^27^ ^,^ ^67^ Amapá^30^ and Roraima,^70^ as well as in the Peruvian Amazon^15^ ^,^ ^110^ where the proliferation of fish farms has created excellent conditions for the increased in abundance of *An. darlingi* and the risk for malaria transmission. It was not possible to analyze the significance of differences of physicochemical characteristics among water body types given the relatively low number of replicates.

An important characteristic of larval habitats is the presence of debris, floating and emergent vegetation as well as margins covered with grasses, providing food for larvae, refuges that protects from predators, and favorable conditions for oviposition.^105^ ^,^ ^111^ ^,^ ^112^ ^,^ ^113^ The dbRDA analysis showed that in general *An. darlingi* and the other anophelines species were negatively associated with various predacious aquatic insect families in the streams (Fig. 5B), except a positive relation between *An. darlingi* and the Nepidae family of Heteroptera, which might be due to only three specimens being collected.

The study of aquatic insects in the Neotropics as predators of mosquito larvae is scarce. There are some studies conducted under laboratory conditions as well as in micro-reservoirs such as artificial containers and phytothelmata showing that predation can control mosquito populations.^114^ Studies conducted in Mexico showed that the abundance of *An. albimanus* was lower in larval habitats with predators such as members of the order Heteroptera.^115^ During the present study, a rich fauna of aquatic insects representing 19 families and five orders were collected, including various predators of mosquito larvae.^55^ ^-^ ^63^ ^,^ ^114^ Nevertheless, the temporal distribution of anophelines is different from that of other aquatic insects. Anophelines are early colonizers of water bodies of the transition period from the rainy to dry season while predators are late colonizers, more abundant in the late rainy season.^51^ On the other hand, the development time of anopheline immature stages is much shorter than that of the predators.^47^ In relation to predators of Amazonian anophelines, it is suggested that only members of the families Noctonectidae and Veliidae (Heteroptera) could be of importance since they feed on small insects that rest on the water surface.^116^ The role of predators in the present study as determinants in the variation of anopheline larval abundance is doubtful. Other factors, such as climate, trophic and physicochemical characteristics of water bodies, might have a greater influence on the temporal and spatial abundance of *An. darlingi* and other anophelines in PVH.

Several studies have documented the influence of rainfall on mosquito larvae abundance.^18^ ^,^ ^19^ ^,^ ^25^ ^,^ ^100^ ^,^ ^117^ Depending on the *Anopheles* species and the type of larval habitat, this relation might be negative or positive. The temporal distribution of *An. darlingi* and associated species in PVH was significantly determined by rainfall and river level, independently of the type of permanent larval habitat. Furthermore, there was clearly a species succession which somehow reduces inter-specific competition: the abundance peak of *An. braziliensis* occurred two months after the peak of rainfall, whereas the peak for *An. triannulatus s.l* and *An. peryassui* occurred a month later (three months after the peak of rain), and *An. darlingi* peaks four months later. In general, the abundance of anophelines is higher in the transition period rainfall-drought and in the dry season (Fig. 3). Similar results were reported for *An. darlingi* larval abundance in Roraima,^31^ the Peruvian Amazon,^15^ southern Venezuela,^18^ and Belize;^118^ whereas Arcos et al.^117^ indicated an increase in the abundance of *An. darlingi*, *An. triannulatus s.l.* and *An. nuneztovari s.l.* during the rainy season in fishponds in Manaus. The increase in rainfall may produce several temporal larval habitats but also might have a negative effect producing the flush of larvae in habitats such as rivers and streams,^71^ ^,^ ^100^ resulting in the reduction of the adult population.^25^ The level of the Madeira River has a significant effect on anopheline’s abundance in PVH. The peak of precipitation precedes the peak river level (Fig. 1). The river level also showed a significant influence on the anopheline’s abundance in a successional manner where the peak of *An. braziliensis* occurred with a lag of one month, two months for *An. peryassui*, three for *An. triannulatus*, and five for *An. darlingi* (Table III).

Several studies have demonstrated the influence of climate on the abundance of *Anopheles* mosquitoes and on malaria in Brazil,^119^ ^,^ ^120^ ^,^ ^121^ ^,^ ^122^ and around the world.^65^ ^,^ ^123^ ^,^ ^124^ ^,^ ^125^ ^,^ ^126^ Studies in Brazil,^120^ ^,^ ^121^ ^,^ ^122^ Peru^127^ and Venezuela^65^ confirm the influence of river level on the incidence of malaria, with peaks after river floods. Most studies on climate, mosquito abundance and malaria incidence focused on adult females, since no correspondence was found between the abundance of adults and immature stages, probably because each life stage has different factors that influence mortality besides the duration of the aquatic cycle. In the present study, several malaria models were developed. Accordingly, water temperature variation was not significant and did not influence the mosquito immature populations. Significant variables were found only for relative humidity when *An. darlingi* was included in the model, and river level when all mosquito species were included. The models proposed in the present study can be used to predict an increase in the number of malaria cases in PVH.

Based on the results of the present study, larval source management of permanent habitats seems a feasible intervention to reduce vector populations and reduce transmission in PVH. Some studies have been conducted in the Brazilian Amazon using biolarvicides for the control of anopheline populations with variable results.^128^ ^,^ ^129^ ^,^ ^130^ Galardo et al.^130^ showed promising results with the use of *Bacillus sphaericus* in mining sites in Amapá. The abundance of immatures of *An. darlingi* were reduced throughout the 52-weeks study and adult populations were reduced during the rainy season. Other studies in the region have shown the effectiveness of *B. sphaericus* in reducing the abundance of *An. darlingi* larvae in fishponds in Peru,^131^ *An. albimanus* in ponds in Colombia^132^ and *An. triannulatus*, *An. nuneztovari s.l*., *An. darlingi*, and *An. albitarsis s.l.* in lagoons in southern Venezuela.^133^ Furthermore, Moreno et al.^134^ showed that there was also a significant reduction in the incidence of malaria cases in gold mining areas. All the reported studies show that the product must be applied every four-nine weeks, and that requirement might make it unsustainable for vector control programs.

A key characteristic of permanent larval habitats in PVH was the presence of marginal vegetation, which provides food and refuge to mosquito larvae. A community-based program for the regular removal of vegetation of habitats such as lagoons, dams and fishponds might be cost-effective and serve as a long-term solution for the elimination of larval habitats.^76^ ^-^ ^135^

In accordance with the objectives of the Brazilian National Plan for the Elimination of Malaria,^12^ the present study provides relevant data on key factors for permanent larval habitats in PVH for the surveillance of *An. darlingi* and other potential vectors, as well as a Negative Binomial model based on immature mosquito abundance and climate variables (Relative Humidity and River Level) for predicting increases in the numbers of malaria cases.
