# Supplementary material for: Characterization of larval habitats of Anopheles (Nyssorhynchus) darlingi and associated species in malaria areas in western Brazilian Amazon
Source: Mem Inst Oswaldo Cruz. 2024 Oct 7;119:e240116. doi: 10.1590/0074-02760240116 (PMC11458182; doi:10.1590/0074-02760240116)
Supplement: Supplementary file 1 [file 1678-8060-mioc-119-e240116-s.pdf]

TABLE I

Rainfall (mm), mean air temperature (ºC), relative humidity (%), and Madeira River level (m).
Porto Velho, Rondônia, Brazil. March 1998 - May 1999[37]

Date	Rainfall (mm)	Temperature (ºC)	Relative humidity (%)	Madeira River level (m)
Jan 98	368.6	26	83	11.42
Feb 98*	-	-	-	-
March 98	331.5	26.5	93.4	14.62
April 98	275.5	29	73	14.77
May 98	117.3	28	68	11.32
June 98	85	29	73	7.66
July 98	6.5	25	69	5.17
Aug 98	49.2	30	72	3.55
Sept 98	30	31	64	3.32
Oct 98	80.8	31	73	4.37
Nov 98	184.2	30	74	8.42
Dec 98	271.3	31	73	11.23
Jan 99	412.9	30	75	12.88
Feb 99	258.4	31	71	14.72
March 99	263.8	31	79	14.82
April 99	267.4	29	74	14.54
May 99	200.1	28	75	11.72

*Data not available.

CC BY

TABLE II

Number of scoops per larval habitat per month for 14 months (April 1998 - May 1999). Porto Velho, Rondônia, Brazil

Code	Type	Location	Number of scoops/month	Total number scoops (14 months)
2	Stream	Est Penitenciária	150	2,100
33	Stream	Est Penitenciária	309	4,326
46	Stream	Col 21 de Abril	171	2,394
56	Stream	Col 21 de Abril	115	1,610
92	Stream	Col Viçosa	160	2,240
98	Stream	Col Viçosa	90	1,260
114	Stream	ASBERON	116	1,624
114-A	Stream	San Antonio	135	1,890
115	Stream	Br 364 km 14	344	4,816
116	Stream	Uberada	210	2,940
124	Stream	Mato Grosso	146	2,044
126	Stream	Santa Clara	102	1,428
128	Stream	Br 364 km 18	170	2,380
132	Stream	Br 364 km 44	87	1,218
137	Stream	Jacy-Paraná	195	2,730
TOTAL			**2,500**	**35,000**
52	Stream+lagoon	Col 21 de Abril	180	2,520
55	Stream+lagoon	Col 21 de Abril	144	2,016
TOTAL			**324**	**4,536**
95	Stream+dam	Col Viçosa	174	2,436
113	Stream+dam	Br 364 km 8	166	2,324
TOTAL			340	4,760
96	Fishpond	Col Viçosa	**138**	**1,932**
112	Lagoon	Br 364 km 8	**151**	**2,114**

TABLE III

Monthly anopheline density* (larvae/scoop) in each sampled type of larval habitat in Porto Velho, Rondônia, Brazil. April 1998 - May 1999

Date	Stream (N = 15)	Lagoon (N = 1)	Fishpond (N = 1)	Stream + lagoon (N = 2)	Stream + dam (N = 2)
April 1998	0.12 - 6.20	1.10	1.84	0.25	2.32 - 5.86
May 1998	1.01 - 4.65	2.34	4.14	2.42	2.27 - 5.47
June 1998	0.78 - 2.30	2.54	1.54	2.07	1.48 - 3.94
July 1998	0.54 - 4.81	5.15	0.60	3.65	2.64 - 5.20
Aug 1998	0.00 - 5.95	1.87	1.66	1.83	1.90 - 2.15
Sept 1998	0.05 - 3.01	0.88	0.63	0.23	0.95 - 1.02
Oct 1998	0.01 - 2.23	0.91	0.17	1.06	0.17 - 0.89
Nov 1998	0.01 - 9.15	2.47	0.23	0.77	0.04 - 0.47
Dec 1998	0.01 - 1.59	4.12	0.05	0.32	0.04 - 0.34
Jan 1999	0.01 - 2.38	1.65	0.01	0.17	0.06 - 5.90
Feb 1999	0.01 - 1.50	1.51	0.08	0.22	0.33 - 1.21
March 1999	0.03 - 1.41	0.04	0.17	0.11	0.22 - 0.30
April 1999	0.10 - 3.04	0.25	1.10	0.90	0.12 - 1.13
May 1999	0.20 - 2.61	0.71	1.82	0.40	0.71 - 0.84

*Range of larvae/scoop collected per month.

TABLE IV

Correlation coefficients of selected variables for distance-based redundancy analysis (dbRDA) significance axis for 15 larval habitats (streams). The components are the environmental variables and temporal variables associated to *Anopheles* spp density. Porto Velho, Rondônia, Brazil. April 1998 - May 1999

	Axis	
Variable	I	II
Silica	+0.04	+0.08
Chlorides	-0.09	-0.04
Dissolved Oxygen (DO)	+0.17	+0.16
Dissolved Carbon Dioxide (DCO_2)	+0.17	+0.20
Calcium carbonate	+0.10	+0.42
Ammonia	+0.15	-0.27
Alkalinity	+0.04	+0.16
Sulphates	+0.18	-0.53
Nitrate	+0.34	-0.36
Phosphorus	-0.26	-0.75
pH	-0.06	0.01
Water temperature	-0.14	+0.31
Shade	-0.08	-0.61
Depth	-0.06	+0.41
April 1998	+0.08	+0.68
May	+0.39	+0.77
June	+0.38	+1.19
July	+0.11	+0.45
August	+0.16	+0.48
September	+0.59	+0.49
October	-0.24	+0.73
November	-0.22	+0.73
December	+0.24	+0.27
January 1999	-0.55	+0.22
February	-0.47	+0.22
March	-0.29	+0.31
April	-0.22	+0.41

TABLE V

Correlation coefficients of the selected variables (aquatic insect families) by the distance-based redundancy analysis (dbRDA) significance axis for 15 larval habitats (streams)

	Axis	
Variable	I	II
Belostomatidae	+0.06	-0.09
Corixidae	-0.13	+0.48
Notonectidae	+0.18	+0.20
Naucoridae	+0.37	+0.02
Pleidae	-0.47	-0.14
Veliidae	-0.17	-0.07
Gerridae	+0.17	-0.06
Hydrometridae	+0.69	+0.24
Nepidae	-0.41	+0.64
Trycorithidae	+0.48	-0.13
Libellulidae	-0.19	-0.23
Coenagrionidae	+0.05	-0.38
Noteridae	+0.38	+0.08
Dytiscidae	+0.16	+0.20
Scirtidae	-0.09	+0.47
